# Evidence-Based Perioperative Medicine comes of age: the Perioperative Quality Initiative (POQI): The 1st Consensus Conference of the Perioperative Quality Initiative (POQI)

**DOI:** 10.1186/s13741-016-0055-y

**Published:** 2016-10-13

**Authors:** Timothy E. Miller, Andrew D. Shaw, Michael G. Mythen, Tong J Gan, For the Perioperative Quality Initiative (POQI) I Workgroup

**Affiliations:** 1 Division of General, Vascular and Transplant Anesthesia, Duke University Medical Center, Durham, NC 27710 USA; 2 Department of Anesthesiology, Vanderbilt University Medical Center, Nashville, TN USA; 3 UCL/UCLH National Institute of Health Research Biomedical Research Centre, London, UK; 4 Department of Anesthesiology, Stony Brook University School of Medicine, Stony Brook, NY USA

**Keywords:** Enhanced recovery, Enhanced recovery pathway, Colorectal surgery, Analgesia, Fluid management, Infection, Measurement, Quality

## Abstract

The 1st POQI Consensus Conference occurred in Durham, NC, on March 4–5, 2016, and was supported by the American Society of Enhanced Recovery (ASER) and Evidence-Based Perioperative Medicine (EBPOM). The conference focused on enhanced recovery for colorectal surgery and discussed four topics—perioperative analgesia, perioperative fluid management, preventing nosocomial infection, and measurement and quality in enhanced recovery pathways.

## Editorial

The population of patients undergoing elective surgery is expanding. It is estimated that worldwide, more than 230 million surgical procedures occur each year (Weiser et al. 2008). An increasing proportion of these patients, as life expectancy increases, are likely to be high-risk and elderly patients with multiple co-morbidities who present particular challenges to anesthesiologists, surgeons, nursing, and other perioperative care providers.

Despite improvements in surgery and anesthesia, approximately one in five patients experience a complication after major surgery (Ghaferi et al. 2009). Complications increase short-term costs and long-term mortality, as well as reduce functional capacity and quality of life (Khuri et al. 2005; Finlayson et al. 2012).

Perioperative complications can be directly caused by surgery or anesthesia but are more commonly related to or exacerbated by the perioperative care processes that occur during the patient’s hospital stay. The optimum perioperative management of patients requires input from a multidisciplinary team.

Perioperative medicine is a term that has been used to describe patient-centered care throughout the surgical pathway and has recently been defined as “a patient-focused, multidisciplinary, and integrated approach to delivering the best possible health care throughout the perioperative journey from the moment of contemplation of surgery until full recovery” (Grocott and Mythen 2015).

Much of perioperative medicine occurs outside of the operating room. Preoperatively, key elements of perioperative medicine are risk evaluation, management of long-term health problems, prehabilitation, lifestyle modification, and shared decision-making. Postoperatively, the appropriate level of care should be targeted to not only achieve functional recovery as quickly as possible, manage long-term co-morbidities, and avoid complications whenever possible but also rescue appropriately when they occur. This care should continue after hospital discharge until full recovery.

Many aspects of perioperative medicine are encompassed in enhanced recovery pathways (ERPs) for major surgery, which are standardized, evidence-based multidisciplinary care pathways to improve outcomes after major surgery (Miller et al. 2014). In many ways, perioperative medicine builds on the success of ERPs.

As perioperative medicine is an emerging specialty, there is a need for consensus material from which to guide practice. The Perioperative Quality Initiative (POQI) brings together multidisciplinary groups of experts from different backgrounds, specialties, and nations to review and debate the current evidence. The POQI strategy is to follow the rules of evidence-based medicine by first developing a critical analysis of the published evidence available for the issues relevant to the conference topic. The ultimate goal is to generate consensus statements that describe, classify, and interpret the available data and offer recommendations for patient care. In addition, each POQI group will aim to identify unanswered questions and areas of clinical and laboratory research that require priority attention.

## 1st POQI conference

The 1st POQI Consensus Conference occurred in Durham, NC, on March 4–5, 2016, and was supported by the American Society of Enhanced Recovery (ASER) and Evidence-Based Perioperative Medicine (EBPOM). The conference focused on “enhanced recovery for colorectal surgery”. The four discussion topics chosen were as follows:

1. Perioperative analgesia—how can we best manage pain within an ERP for colorectal surgery
2. Perioperative fluid management—how can we best manage fluid within an ERP for colorectal surgery
3. Preventing nosocomial infection—how can we best prevent nosocomial infection within an ERP for colorectal surgery
4. Measurement and quality—how can we measure the of quality of care within an ERP for colorectal surgery

The investigators in each work group are listed in Appendix. We believe that these topics are important for successful implementation of ERPs within colorectal surgery. Within each topic, we have attempted to make recommendations that are both useful and practical for clinicians. While ASER supported the conference, and the work group members were asked to focus on ERPs in the USA, we believe that many of the recommendations will apply to ERPs in any country.

## POQI process

The POQI conference process is inspired by and developed from the methodology used by the Acute Disease Quality Initiative. This has been described in detail previously (Kellum et al 2008), and consists of three stages: pre-conference planning, conference, and post-conference.

During the pre-conference phase, the POQI board and conference directors select topics that will benefit from the POQI process of detailed discussion and analysis of the evidence. The reasons to choose a topic are multifactorial but are broadly based on an apparent need for a consensus statement from a group of international experts to offer recommendations for patient care. Typically, three to four independent but related topics are discussed at each POQI conference.

Work groups are then assembled to tackle each topic. The work group consists of a chair, co-chair, and several delegates. Delegates may or may not be an expert on the assigned topic, but all topics are presented to the group at large, and all delegates are able to contribute to the preparation of each manuscript during the POQI conference.

The pre-conference work is key to a successful POQI conference. Each work group thoroughly reviews the literature, generates a bibliography of relevant studies, and identifies a list of key questions to be addressed in the final manuscript.

Each work group:

- Assesses the current of the state of knowledge and classifies the evidence according to evidence-based medicine principles
- Identifies areas of inadequate knowledge, unanswered questions, and prioritized areas of research
- Describes appropriate methodological strategies by which these issues could be addressed in the future

In many cases, a workgroup ends the pre-conference activity with a draft manuscript.

The POQI conference itself is an intensive 2-day interactive conference where delegates are encouraged to debate and question the key issues in each topic. The conference agenda is divided into plenary sessions, where the pre-conference and later conference findings and deliberations are presented, debated, and refined, and breakout sessions, where work groups address the issues in their assigned topic area. The Conference Directors’ role is to circulate among the breakout groups to direct discussion where appropriate and to facilitate and moderate the plenary sessions.

During each plenary session, a different workgroup member presents the results of discussion during the breakout session to the entire group, revising each key statement until delegates agree on a final version. If agreement cannot be reached, this is documented in the manuscript.

Post-conference, each workgroup finalizes a consensus statement on their topic for publication in a peer-reviewed journal. The conference directors, whose names are included as co-authors, must approve the workgroup manuscripts. The final manuscripts are circulated to all delegates for comment and revision before submission for publication. The manuscripts and key figures are made available on the POQI website, poqi.us.

## Conclusion

We believe the five manuscripts in this series will be a valuable addition to the literature and provide guidance as to the current state of the literature regarding ERPs for colorectal surgery. We have attempted to provide practical consensus statements and recommendations to guide practice where consensus exists and to identify areas of inadequate knowledge and unanswered questions.

## Appendix

### POQI I investigators listed by workgroup

POQI I conference directors

Timothy E Miller, Department of Anesthesiology, Duke University Medical Center, NC, USA. Andrew D Shaw, Department of Anesthesiology, Vanderbilt University Medical Center, Nashville, TN, USA. Michael (Monty) G Mythen, Department of Anaesthesia, University College London, London, UK. Tong J Gan, Department of Anesthesiology, Stony Brook University School of Medicine, NY, USA.

Group A—analgesia

Matthew D. McEvoy, Department of Anesthesiology, Vanderbilt University Medical Center, Nashville, TN, USA (chair). Michael J. Scott, Department of Anaesthesia, Royal Surrey County NHS Foundation Hospital, Surrey, UK (co-chair). Deborah Gordon, RN, Department of Anesthesiology and Pain Medicine, University of Washington. Stuart Grant, Department of Anesthesiology, Duke University Medical Center, NC, USA. Julie K.M. Thacker, Division of Advanced Oncologic and GI Surgery, Duke University Medical Center, NC, USA. Christopher L. Wu, Department of Anesthesiology, The Johns Hopkins University School of Medicine, MD, USA.

Group B—fluids

Robert H. Thiele, Departments of Anesthesiology and Biomedical Engineering, University of Virginia School of Medicine, VA, USA (chair). Karthik Raghunathan, Department of Anesthesiology, Duke University Medical Center, USA (co-chair). C S Brudney, Department of Anesthesiology, Duke University Medical Center, USA. Dileep N Lobo, Division of Gastrointestinal Surgery, Nottingham University Hospitals and University of Nottingham, Nottingham, UK. Dr. Daniel Martin, Royal free Perioperative Research Group, Royal Free Hospital, London, UK. Anthony Senagore, Department of Surgery, University of Texas-Medical Branch at Galveston, Galveston, TX, USA. Maxime Cannesson, Department of Anesthesiology and Perioperative Medicine, University of California Los Angeles, CA, USA

Group C—infection

Stefan Holubar, Department of Surgery, Dartmouth-Hitchcock Medical Center, NH, USA (chair). Traci Hedrick, Department of Surgery, University of Virginia School of Medicine, VA, USA (co-chair). John Kellum, Department of Critical Care Medicine, University of Pittsburgh School of Medicine, Pittsburgh, PA, USA. Ruchir Gupta, Department of Anesthesiology, Stony Brook University School of Medicine, NY, USA. Mark Hamilton, Department of Anaesthesia, St. George’s Hospital and Medical School, London, UK.

Group D—outcomes

Ramani Moonesinghe, Department of Anaesthesia, University College London, London, UK. (chair). Mike Grocott, Department of Anesthesia and Critical Care Medicine, University of Southampton, UK (co-chair). Elliott Bennett-Guerrero, Department of Anesthesiology, Stony Brook University School of Medicine, NY, USA. Tom Hopkins, Department of Anesthesiology, Duke University Medical Center, NC, USA. Roberto Bergamaschi, Department of Surgery, Stony Brook University School of Medicine, NY, USA. Stuart McCluskey, Department of Anesthesia, University of Toronto, ON, Canada.

## Abbreviations

ASER: American Society of Enhanced Recovery
EBPOM: Evidence-Based Perioperative Medicine
ERP: Enhanced recovery pathway
POQI: Perioperative Quality Initiative
